# Oral chromoblastomycosis: a case report

**Published:** 2012-03

**Authors:** Fatemi MJ, Bateni H

**Affiliations:** 1 Plastic & Reconstructive Surgery, Department of Plastic & Reconstructive Surgery and Burn Research Center, Hazrat Fateme Hospital, Tehran University of Medical Sciences, Tehran, Iran; 2 Plastic & Reconstructive Surgeon, Mehr private hospital, Tehran, Iran

**Keywords:** Chromoblastomycosis, palatal reconstruction, radial forearm flap, oronasal competence, fasciocutaneous free flap

## Abstract

A chronic fungal infection in tropical regions, chromoblastomycosis is caused by dematiaceous fungi, the form-family of Fungi Imperfecti, usually affecting one lower limb at the site of a trauma but sometimes involving other areas of the body including head & neck. In this article, we report the case of a rare primary chromoblastomycosis of the palate and chest in a 27-year-old man who was successfully treated with surgical resection and combined drug therapy, and eventually free tissue transfer reconstructive surgical procedure to cure the palatine defect.

## INTRODUCTION

It is generally accepted that chromoblastomycosis usually affects one leg or foot. In some rare instances the disease begins on the hand or wrist and involves the entire upper extremity. It may also begin on the face. Chromoblastomycosis is caused in man by dematiaceous fungi (1–3). It begins as a small papule or warty growth and slowly spreads by the growth of satellite lesions (1).The affected area is usually swollen and there is a slow progression of the disease. Plaque-like and cicatricial types of lesions also occur (4). The lesions that may or may not ulcerate are characterized by round, brown bodies^5^ that reproduce by equatorial splitting (6). The infection is commonly seen among barefooted farm laborers, (2) predominantly in males in the 20-50-year age range (2). It has been recognized in different countries (7, 8). Our report describes an unusual case of oral chromoblastomycosis which was treated successfully with antifungal agents and surgical intervention.

## CASE REPORT

A 27-year-old, otherwise healthy normal male presented to the reconstructive surgery clinic of Hazrat Fateme hospital. His problem first appeared about 11 years ago, as a small pink lesion on the hard palate and a simultaneous scaly papule on the anterior chest. Several hospital admissions and therapies including antibiotics did not have any effect on the disease. The lesions grew gradually interfering with oral function and finally the diagnosis was confirmed through pathologic and mycological studies. The biopsy results are discussed later. All other test results including blood biochemistry, hematology, sexually transmitted disease (STD) serology, urine analysis and microbiology, and stool exam were within normal limits. Moreover, intact cell mediated immunity was revealed. Imaging studies showed the extent of mass growth and the involvement of surrounding bone. The mass was resected sacrificing the involved soft tissue and bone, and the palatal defect was covered by means of local tissue, only to fail. The patient received Amphotericin B and Itraconazole according to a protocol provided by clinical mycologists of the department of infectious diseases (4, 9). Finally, after confirming the disease free margins of the defect, the decision to employ free tissue transfer was made. To cover this 38 by 42 mm three-dimensional defect in the palate, a well vascularized thin tissue providing viable skin was needed. Along the length of the pedicle was an issue to facilitate microsurgical transplantation. Thus, a free radial forearm fasciocutaneous flap was chosen (10).

**Fig. 1:**
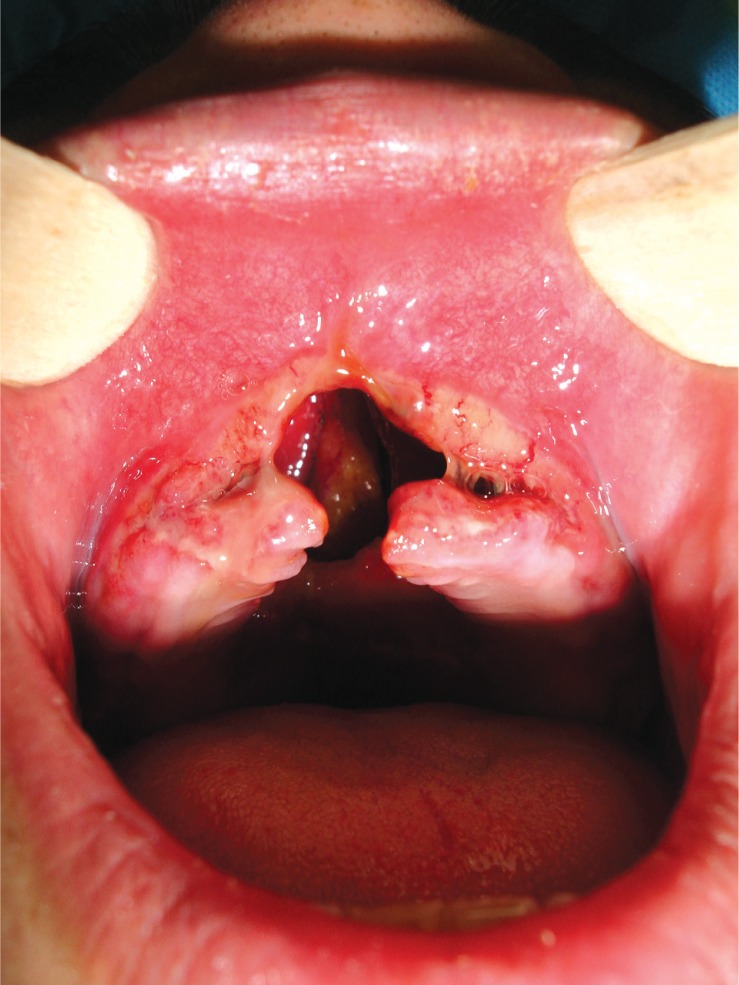
The appearance of the palatal oral cavity defect.

**Fig. 2:**
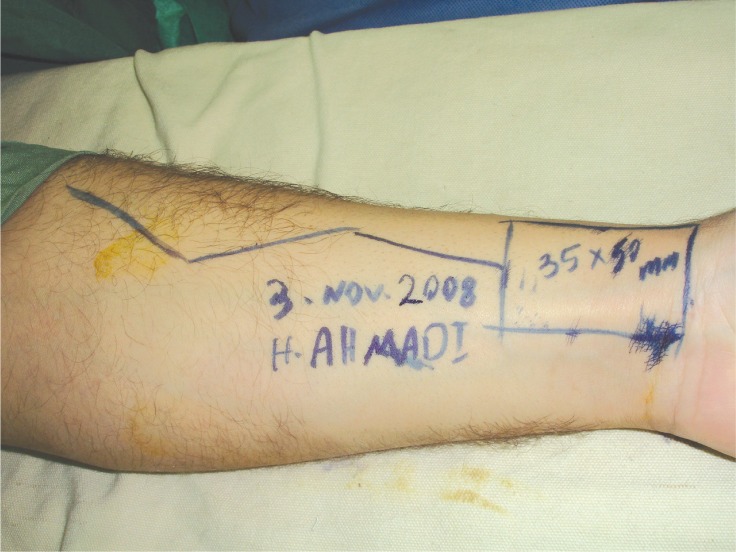
Design of the radial forearm flap.

The patient underwent surgery once the necessary tests were run and the patency of the ulnar artery and deep palmar arch was confirmed preoperatively. The flap was dissected on the nondominant limb in a distal to proximal direction on its radial artery pedicle and venae comitantes. After preparation of the Facial vessels as the recipient vessels, the flap was harvested and transplanted. The nasal lining was restored by split thickness skin graft. The patient received postoperative care and was discharged after the donor site dressing and immobilizing splint were removed, and scheduled for several office visits.

**Fig. 3:**
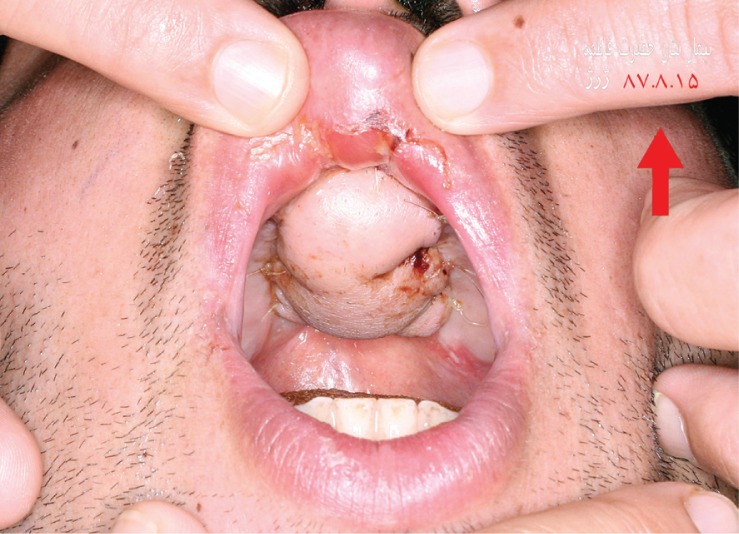
The post-operative view of healed palate.

**Fig. 4:**
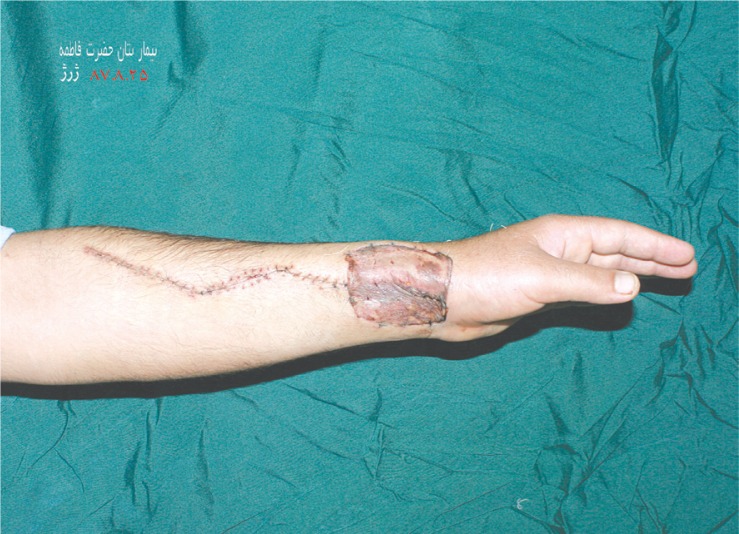
The donor site on the non-dominant forearm.

### Pathologic and mycological findings

The histological finding in the biopsy specimen was that of a granulomatous reaction characterized by formation of pseudotubercles containing giant cells, and a focal round cell infiltration in the superficial portions of the specimen. The fungi appeared in clusters of brown, spherical cells with thick, dark cell walls and coarsely granular pigmented protoplasm. The cultural characteristics were also studied. The colonies produced black, slow growing, heaped-up colonies and the *Cladosporium* type of sporulation was noted.

The last specimens obtained before the final reconstructive procedure showed eosinophil rich acute inflammation without any fungal infection. Cultures obtained as the reference method were all negative.

## DISCUSSION

Chromoblastomycosis usually begins on one lower extremity. The small lesions may resemble common warts (11). The cicatricial types are formed by nodules and spread peripherally (12, 13). Healing with sclerosis takes place at the center, at times associated with keloid formation. Lymphadenopathy may occur as a result of secondary bacterial complication. There are five dematiaceous fungi known as the main causative agents: *Cladophialophoracarrionii, Fonsecaeapedrosoi, F.compacta, Phialophoraverrucosa*, and *Rhinocladiella aquaspersa* (1, 5). The disease occurs 20 times more commonly in men than in women (14).There is slow progression and the disease may take many years to develop fully.^4^ Usually the disease process remains localized to one lower extremity, however, there are reports of various clinical involvements, even in the central nervous system, with or without associated skin lesions. Metastases through the blood stream are rare, but have been documented (15). The involvement of oral cavity presented in our case is extremely rare. Our patient had no predisposing factors including advanced age, poor social condition or hygiene, diabetes, vascular occlusive disease, alcoholism, mental/psychiatric illness, or physical disability.

Despite medical and surgical therapy, cure of chromoblastomycosis infection is difficult and recurrences are frequent as in our case. As yet, no controlled therapeutic trials have been reported, so there is no medication or combination of medications considered as treatment of choice. Antifungals are needed to be given for at least 6-12 months, often combined with physical treatments such as surgery, cryotherapy and thermotherapy. Cure rates range from 15% to 80% (16).

The essential pathologic changes are characterized by a granulomatous reaction (17) and the fungus occurring in the form of spherical cells (1) not reproducing by budding. Septate forms are occasionally encountered, this being the characteristic tissue morphology of chromoblastomycosis, differentiating it from other fungal infections (8).

The oral cavity contents perform important functions including speech, mastication, deglutition, maintenance of oronasal competence, salivation, and early digestion. Hence, oral cavity reconstruction should aim to restore both form and function. The palate divides the upper airway into two parts, oral and nasal, allowing speech and aiding in deglutition. The oral cavity reconstruction has been revolutionized by microvascular surgery (18). The reconstructive surgeon faces a variety of flaps available to find a match to reconstruct a defect (19). The free radial forearm flap may be suitable for covering many intraoral defects. It is an excellent choice for oral lining restoration when bulk is not required. The most notable advantages of this flap include constant anatomy of the radial artery, its large caliber, extensive length of the pedicle, (20) large territory, easy dissection, potential for creating a sensate flap, (21) thin malleable tissue, and reliability (22). The most important disadvantage of the flap relates to its donor site morbidity (23) which can be minimized if necessary precautions and modalities are taken into consideration (24).
